# Secondary demyelination after stroke: Glial cell crosstalk

**DOI:** 10.1016/j.ibneur.2026.02.004

**Published:** 2026-02-04

**Authors:** Ruonan Cao, Chaoran Liu, Zhihui Liu, Wenjing Ou

**Affiliations:** a Baotou Clinical Medical College of Inner Mongolia Medical University, Baotou, China; b Department of Anesthesiology, Baotou Central Hospital, Baotou, China; c Center College for Clinical Medicine of Baotou Medical College, Baotou, China; d Department of Medical Records, Baotou Central Hospital, Baotou, China

**Keywords:** Stroke 1, Secondary demyelination 2, Astrocyte 3, Lipocalin-2 4, Microglia 5, Oligodendrocyte 6

## Abstract

Neuroglial cells serve as the myelinating cells in the central nervous system and are essential for axonal integrity and function. Following a stroke, progressive loss of myelin occurs in white matter regions distal to the infarct core, contributing to cognitive decline and even dementia. Recent research has revealed that the central mechanism involves a cascading response triggered by disruption of the astrocyte-microglia-oligodendrocyte axis. Astrocyte-secreted lipocalin-2 acts as a key driver of myelin degradation and axonal energy crisis by inducing iron influx, triggering oxidative stress, and causing mitochondrial dysfunction. An imbalance in microglial subpopulations, along with oligodendrocyte apoptosis, further exacerbates demyelination. Although no therapies are currently approved, novel strategies targeting lipocalin-2 blockade, mitochondrial repair, and immune modulation offer new hope for preserving white matter function.

## 1 Introduction

**Fig. 1:**
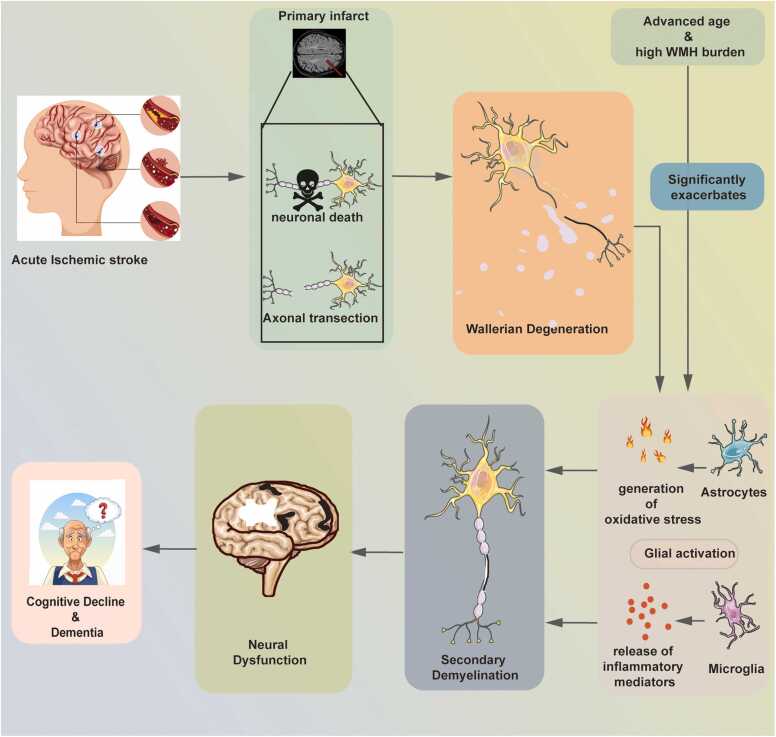
Schematic diagram of the core mechanism of secondary demyelination mediated by neuroglial cells after stroke. Figure legend: The schematic illustrates the pathological cascade following an acute ischemic stroke. The initial infarct leads to Wallerian degeneration, which subsequently activates microglia and astrocytes. These activated glial cells release inflammatory mediators and ROS, driving secondary demyelination in white matter areas remote from the original infarct. This demyelination process ultimately results in neural circuit dysfunction and cognitive decline. The entire process is potentiated by risk factors such as advanced age and a high burden of WMH. Abbreviations: WMH, white matter hyperintensity; ROS, reactive oxygen species.

Stroke is defined as a group of diseases in which sudden rupture or blockage of cerebral blood vessels interrupts the blood supply to the brain, resulting in tissue damage (Sacco et al., 2013). While stroke severity is predominantly linked to lesions in bilateral subcortical and left cortical regions, a high burden of white matter hyperintensity, in turn, significantly influences how lesions in the left insular and temporoparietal regions affect severity (Bonkhoff et al., 2022). Secondary demyelination is a form of white matter pathology characterized by myelin loss due to damage to other neural structures. In the context of stroke, it refers to the progressive myelin degradation that occurs days to months after ischemia-reperfusion in white matter regions distant from the initial infarct core. Following a stroke, Wallerian degeneration occurs in focal cortical regions as well as in non-ischemic areas such as the corpus callosum and striatum—a process which involves amyloid precursor protein accumulation, neuroglial cell activation, glial scar formation, axonal injury, and subsequent distal myelin degradation (Zuo et al., 2019). Post-stroke secondary demyelination is linked to neurological dysfunction (Xin et al., 2024). Furthermore, its progression and long-term outcomes are influenced by age-related increases in oxidative stress and inflammatory mediators (Poirier et al., 2025). Cohort studies in patients with ischemic stroke or cerebrovascular disease have demonstrated that secondary demyelination is an independent predictor of cognitive decline. Specifically, even after adjusting for confounders like age, education, and infarct volume, individuals with extensive demyelination show a markedly higher risk of global cognitive decline or dementia (Abel et al., 2020, Dao et al., 2021). Evidence suggests that neuroglial cells play a central role in post-stroke secondary demyelination. Notably, as a rapidly induced acute-phase protein, lipocalin-2 (LCN2) functions as a key mediator of post-injury neuroinflammation (Li et al., 2023). Emerging evidence implicates LCN2 not only in regulating iron homeostasis and apoptosis but also as a critical driver of glial activation, positioning it as a key contributor to post-stroke secondary demyelination (Ferreira et al., 2015). Therefore, this review focuses on neuroglial cells. It aims to summarize their contribution to secondary demyelination after stroke and to explore the specific regulatory role of LCN2 in this pathology. (Fig. 1).

## 2 Pathogenesis of secondary demyelination after stroke

### 2.1 Neuroglial crosstalk

**Fig. 2:**
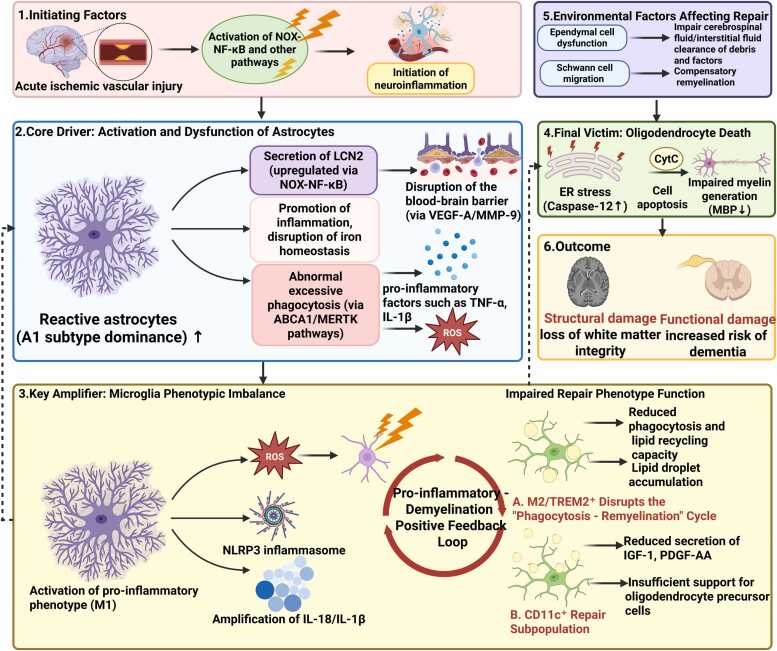
Cascade of Secondary Demyelination After Stroke. Figure legend: This figure illustrates the core pathological mechanisms driving progressive secondary demyelination in white matter distal to the initial infarct following acute ischemic stroke. The cascade is triggered by ischemic injury, which induces neuroinflammation and promotes the activation of astrocytes into a detrimental A1 subtype. These activated astrocytes propagate inflammation through the release of lipocalin-2 (LCN2), aberrant phagocytic activity, and reactive oxygen species (ROS) production. This response forms a feed-forward pro-inflammatory loop with microglia polarized toward an M1 phenotype, collectively exacerbating inflammation and oxidative stress, ultimately leading to oligodendrocyte death and myelin destruction. Concurrently, impaired function or inadequate expansion of reparative microglial subsets (e.g., M2, CD11c⁺), particularly in aging, results in failed clearance of myelin debris and a lack of pro-regenerative signals, representing a critical bottleneck for remyelination failure. The overall microenvironment is further modulated by other cell types. This vicious cycle culminates in progressive white matter demyelination and clinical deficits such as cognitive impairment.

The pathological progression of post-stroke secondary demyelination is initiated by disruption of the astrocyte–microglia–oligodendrocyte axis, triggering a downstream cascade (Lu and Wen, 2024) (Fig. 2). In a mouse model, Liu et al. revealed that acidosis activates a chloride channel, which facilitates chloride influx and engages the Akt/Bax/Caspase-3 signaling pathway to induce neuroglial apoptosis, thereby contributing to secondary demyelination (Liu et al., 2025).

#### 2.1.1 Astrocytes: the central driver of secondary demyelination

**Fig. 3:**
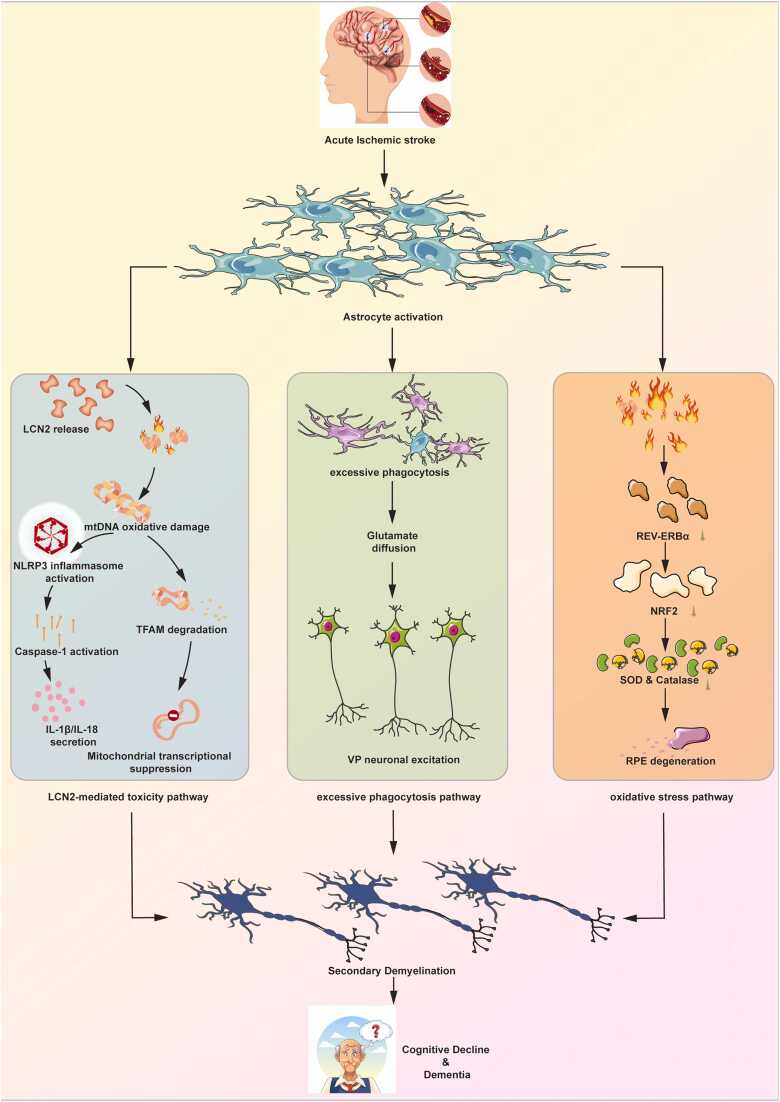
Astrocytes drive core mechanisms of secondary demyelination after stroke. Figure legend: After ischemic stroke, astrocytes activate into reactive astrocytes. Astrocytes drives demyelination via three major pathways: (1) LCN2-dependent toxicity: NOX/NF-κB activation promotes LCN2 secretion, inducing BBB disruption (via VEGFA/MMP-9), iron dyshomeostasis, and neutrophil infiltration. (2) Dysregulated phagocytosis: ABCA1/MERTK-mediated myelin debris clearance becomes pathological, sustaining inflammation via TNF-α/IL-1β. (3)Oxidative stress: ROS production directly damages oligodendrocytes. Collaborative effects lead to progressive demyelination in white matter, causing conduction delay and cognitive decline. Abbreviations: LCN2: Lipocalin-2; BBB: blood-brain barrier; ROS: reactive oxygen species.

A substantial body of evidence from both in vivo and in vitro studies consistently demonstrates that astrocytes are central drivers in triggering and amplifying secondary demyelination after the acute phase of stroke (Coutinho Costa et al., 2023) (Fig. 3). Astrocytes drive progressive secondary demyelination in white matter distal to the infarct core within 7–30 days post-stroke through integrated pathways that include LCN2 secretion, phagocytic activity, and oxidative stress (Wan et al., 2022). This demyelination process leads to clinical deficits including conduction delays, executive dysfunction, and an elevated risk of dementia (Li et al., 2025a). Following ischemic-reperfusion injury, astrocytes become reactive, a state characterized by two key subtypes: the detrimental A1, which promotes neuronal and oligodendrocyte death, and the neuroprotective A2 (Tao et al., 2016). Stroke-induced activation of the NOX–NF-κB pathway in reactive astrocytes upregulates LCN2 (Liu et al., 2022). Functionally, LCN2 knockdown reduces myelin loss by 45 % and improves cognition, while its re-expression restores pathological damage (Xie et al., 2023). A distinct, contributory mechanism is the excessive phagocytosis of myelin debris by astrocytes (Verkhratsky et al., 2023). LCN2 promotes the release of vascular endothelial growth factor A (VEGFA) from astrocytes; VEGFA disrupts endothelial tight junctions and interacts with matrix metallopeptidase 9 (MMP-9), leading to sustained blood-brain barrier (BBB) leakage and damage (Kawa et al., 2025). In parallel, LCN2 induces neutrophil adhesion and impairs BBB signaling, which perpetuates neuroinflammation and disrupts iron homeostasis (Luo et al., 2022). In a focal cortical ischemia model, Wan et al. showed that reactive astrocytes extensively engulf myelin debris via an ABCA1/MERTK phagocytic program—a process which, although initially beneficial for clearing necrotic tissue, becomes detrimental when sustained, causing “secondary myelin damage,” enhancing TNF-α and IL-1β release, and ultimately perpetuating demyelination while blocking remyelination (Wan et al., 2022)

#### 2.1.2 Microglial imbalance

Emerging evidence indicates that post-stroke secondary demyelination results not from oligodendrocyte progenitor cell (OPC) depletion, but from an imbalance in microglial subpopulations (Ji et al., 2024) (Table 1). Excessive polarization of microglia toward the pro-inflammatory M1 phenotype mediates demyelination via the release of inflammatory mediators and reactive oxygen species (Nan et al., 2024). Conversely, the alternatively activated M2 phenotype facilitates remyelination by secreting anti-inflammatory and trophic factors that promote the recruitment, proliferation, and differentiation of OPCs (Han et al., 2024). By phagocytosing damaged myelin and cellular debris, they help suppress secondary immune responses, which in turn ameliorates the inflammatory microenvironment (Han et al., 2024). The CX3CR1/CX3CL1 signaling axis is highly active in microglia. Within 24 h post-stroke, these microglia rapidly polarize toward an M1 phenotype, characterized by elevated expression of iNOS, IL-1β, and TNF-α, and by the release of reactive oxygen species (ROS) and nitric oxide (NO). These ROS directly oxidize lipids in the oligodendrocyte membrane and activate the NLRP3 inflammasome. This activation amplifies the IL-18/IL-1β cascade, establishing a pro-inflammatory and pro-demyelination positive feedback loop (Shi and Yong, 2025). Moreover, activated microglia can directly drive demyelination through CSF1R signaling (Marzan et al., 2021). Secondary demyelination, mediated by microglial imbalance, not only leads to conduction blockade but also independently correlates with post-stroke cognitive impairment (Guo et al., 2022). The specific mechanism underlying this cognitive decline may involve regional desynchronization of microglial activity (Zatcepin et al., 2024). Furthermore, Ronzano et al. identified the Nodes of Ranvier as critical sites for microglia-neuron interactions, which are involved in microglia-mediated remyelination after injury (Ronzano et al., 2021). At these sites, altered potassium flux along axons can influence the regenerative phenotype of microglia and thereby reduce remyelination efficiency (Ronzano et al., 2021). Safaiyan et al. reported high expression of TREM2 in activated microglia after stroke and showed that impaired TREM2-mediated function in disease-associated microglia (DAM) disrupts the scavenger–lipid metabolism axis, which in turn exacerbates myelin loss (Safaiyan et al., 2021). Following stroke, a TREM2⁺/CD11c⁺ disease-associated microglia (DAM) subpopulation appears in white matter (Zhu et al., 2024). Normally, TREM2⁺ DAM clear myelin debris and support lipid recycling; but in TREM2 deficiency, microglia show impaired phagocytosis and accumulate cholesteryl esters. This leads to lipid droplet formation, upregulation of Perilipin-2, reduced TGF-β1 expression, and ultimately, disruption of the phagocytosis–remyelination cycle (Wei et al., 2024, Wang et al., 2023a). Furthermore, remyelination is impaired when the repair-oriented CD11c⁺ microglial response is insufficient. Jia et al. noted that CD11c⁺ microglia appearing at 7 days post-stroke highly express IGF-1 and PDGF-AA to promote OPC proliferation and differentiation; selective depletion of this subpopulation via the CD11c-DTR system consequently reduced remyelination by 55 % (Jia et al., 2023). Notably, aged mice show significantly impaired expansion of this subset, consistent with the increased susceptibility of elderly patients to post-stroke secondary demyelination (Jia et al., 2023).

**Table 1:** Mechanisms of microglial subtype imbalance in post-stroke secondary demyelination.

Time window	Microglial subtype/Marker	Core mechanism	Functional effect	Pathological consequence	Reference
Early Phase (≤24 h)	M1-like polarization^a^	CX3CR1/CX3CL1 axis activation; ↑ iNOS, IL-1β, TNF-α, ROS/NO	NLRP3 inflammasome activation^b^ → IL-18/IL-1β cascade; Lipid oxidation in oligodendrocytes	"Inflammation-demyelination" positive feedback loop	(Ji et al., 2024, Nan et al., 2024, Shi and Yong, 2025)
	CSF1R signaling	Direct activation of demyelination pathways	Accelerated myelin loss	Secondary demyelination initiation	(Marzan et al., 2021)
Subacute Phase (3–7d)	TREM2⁺/CD11c⁺ DAM^c^	Impaired TREM2-mediated lipid metabolism (↓ phagocytosis, ↑ CE accumulation, ↑ PLIN2)	Failed myelin debris clearance; Disrupted lipid recycling → ↓ TGF-β1	Phagocytosis-remyelination cycle interruption; Aggravated demyelination	(Safaiyan et al., 2021, Wei et al., 2024, Wang et al., 2023a)
	CD11c⁺ repair subset	↑ IGF-1, PDGF-AA secretion	Direct promotion of OPC proliferation & differentiation	Critical for remyelination (depletion → 55 % ↓ remyelination)	(Jia et al., 2023)
Chronic Phase	M2-like polarization	Anti-inflammatory & regenerative factor secretion (e.g., TGF-β, growth factors)	OPC recruitment/proliferation/differentiation; Myelin debris clearance; Inflammation resolution	Remyelination promotion; Inflammation microenvironment improvement	(Han et al., 2024)
—	Regional microglial asynchrony	Dysregulated spatial-temporal activity	Impaired neural circuit repair	Independent association with post-stroke cognitive impairment	(Guo et al., 2022, Zatcepin et al., 2024)
—	Node of Ranvier interaction	Altered potassium flux at axonal nodes	Modulates microglial regenerative phenotype	Reduced remyelination efficiency	(Ronzano et al., 2021)
Aging Context	CD11c⁺ subset	Impaired expansion in aged brain	↓ IGF-1/PDGF-AA → reduced OPC activation	Higher susceptibility to demyelination in elderly patients	(Jia et al., 2023)

a Refers to classically activated microglia/macrophages, characterized by the upregulation of inducible nitric oxide synthase (iNOS) and pro-inflammatory cytokines

b A multiprotein complex that, upon activation by cellular damage signals, catalyzes the maturation and release of interleukin-1β (IL-1β) and interleukin-18 (IL-18), serving as a critical platform driving early neuroinflammation.

c Disease-Associated Microglia

#### 2.1.3 Oligodendrocyte damage

Damage to oligodendrocytes can lead to post-stroke demyelination and impaired remyelination, which severely compromises axonal function, structure, metabolism, and survival (Huang et al., 2023). Demyelination is associated with oligodendrocyte apoptosis and downregulation of myelin basic protein (MBP), along with increased expression of the endoplasmic reticulum stress marker caspase‑12 and the release of cytochrome c (Tan et al., 2022). In a study using a distal middle cerebral artery occlusion (MCAO) mouse model, Zhang et al. found that post-MCAO activation of endoplasmic reticulum stress and mitochondrial dysfunction pathways caused oligodendrocyte damage and apoptosis, leading to secondary demyelination throughout the corpus callosum (Zhang et al., 2025). Concurrently, ROS and NO that are released by astrocytes directly target the integrity of mitochondria in oligodendrocytes (Molina-Gonzalez et al., 2023). Altered Ca²⁺ signaling, chronic inflammation, and oxidative stress cause prolonged damage to oligodendrocytes, axons, and myelin, leading to further impairment of white matter integrity and exacerbated demyelination (Nguyen et al., 2021). Multiple studies indicate that reduced glutathione synthesis in astrocytes, along with decreased monocarboxylate transporter function and dysregulated glutamate homeostasis after stroke, subjects the axon–oligodendrocyte unit to sustained oxidative stress and energy failure (Macrez et al., 2016).

#### 2.1.4 The emerging roles of schwann cells and ependymal cells

Conventionally, Schwann cells are restricted to the peripheral nervous system (Salzer et al., 2024). However, accumulating evidence suggests that following severe CNS injury such as stroke, they can enter the lesion site from spinal nerve roots or differentiate from central precursors to participate in remyelination (Bosch-Queralt et al., 2023). Unlike oligodendrocytes, Schwann cells exhibit greater resilience to oxidative stress and can promote axonal regeneration by secreting neurotrophic factors (Frostick et al., 1998). Their presence may thus compensate for oligodendrocyte loss in the ischemic penumbra, although their interaction with CNS glia is not fully understood.

Similarly, ependymal cells lining the ventricular system play a crucial role in cerebrospinal fluid (CSF) homeostasis and metabolic waste clearance (Groh et al., 2024). In the context of secondary demyelination, their dysfunction can impair the clearance of myelin debris and inflammatory cytokines from the interstitial fluid (Groh et al., 2024). Furthermore, these cells have latent stem cell potential; in response to ischemic stimuli, they may proliferate and differentiate into glial scar-forming astrocytes or oligodendrocyte progenitors, thereby influencing the white matter repair environment (Rodriguez-Jimenez et al., 2023).

### 2.2 Key molecular

Studies consistently point to two mutually reinforcing pathways driving post-stroke demyelination: astrocyte-derived LCN2 (a lipocalin superfamily protein) functioning as an upstream trigger, and mitochondrial dysfunction in oligodendrocytes and axons as the downstream effector. Their synergy creates a vicious cycle, leading to myelin loss, axonal energy failure, and cognitive decline (Lin et al., 2025, Wang et al., 2025).

#### 2.2.1 The upstream dominance of LCN2

LCN2, a key mediator of post-stroke demyelination, is secreted by astrocytes and disrupts normal astrocyte-oligodendrocyte interactions, thereby exacerbating demyelination (Cai et al., 2025). LCN2 acts through multiple pathways (Table 2), including the JAK2–STAT3 signaling pathway, the HIF-1α–LCN2–VEGFα axis, and the PKR-like endoplasmic reticulum kinase (PERK) pathway (Laohavisudhi et al., 2024). LCN2 is endocytosed into mature oligodendrocytes via the 24p3R/LRP2 receptor. Once inside the cell, it activates the JNK3/c-Jun apoptotic pathway and suppresses Olig1/Olig2 transcriptional activity, ultimately inhibiting the differentiation of oligodendrocyte precursor cells (OPCs) into myelinating cells (Chen et al., 2020, Zhang et al., 2022, Fields, 2010). Moreover, LCN2 promotes neuroinflammation and cognitive impairment by impairing neuronal NMDA receptor function (Kim et al., 2024). A study in a tMCAO rat model reported that LCN2 mRNA was elevated in astrocytes within the peri-infarct white matter, specifically in those expressing glial fibrillary acidic protein (GFAP⁺, a marker of astrocyte activation), beginning 6 h after reperfusion. Furthermore, LCN2 knockout suppressed iNOS expression (Zhao et al., 2019). By 7 days post-stroke, astrocytes (GFAP⁺) in non-ischemic corpus callosum regions were activated, expressed LCN2, and showed increased phagocytosis of myelin debris (dMBP⁺) (Wan et al., 2022). Conversely, genetic deletion of LCN2 produced a range of protective effects, including increased myelin area, improved white matter integrity, enhanced myelination parameters, and reduced callosal demyelination (Wan et al., 2022). Furthermore, LCN2 facilitates iron entry into oligodendrocytes. This iron influx increases intracellular free Fe²⁺, which induces the Fenton reaction and promotes a surge of ROS (Zhao et al., 2023). Moreover, in conditions like traumatic brain injury and intracranial hemorrhage, LCN2 contributes to blood-brain barrier disruption, increased programmed cell death, and dysregulated iron homeostasis through its interactions with multiple inflammatory cytokines (Zhang et al., 2022).

**Table 2:** Multifaceted mechanisms of LCN2 in driving post-stroke demyelination.

Mechanistic axis	Key molecular events	Cellular targets	Functional consequences	Intervention evidence	Reference
Astrocytic Source	↑ LCN2d secretion by GFAP⁺ astrocytes (6 h post-reperfusion)	Reactive astrocytes	Disrupts astrocyte-oligodendrocyte crosstalk	LCN2 KO → ↓ iNOS expression in peri-infarct white matter	(Cai et al., 2025, Zhao et al., 2019)
OPC Differentiation Suppression	LCN2 endocytosis via 24p3R/LRP2e → JNK3/c-Jun activation; ↓ Olig1/Olig2 activity	Mature oligodendrocytes	Inhibits OPC differentiation into myelin-forming cells	—	(Chen et al., 2020, Zhang et al., 2022, Fields, 2010)
Iron-Mediated Toxicity	LCN2-bound iron influx → ↑ intracellular Fe²⁺ → Fenton reaction	Oligodendrocytes	ROS storm generation → lipid peroxidation & myelin damage	—	(Zhao et al., 2023)
Phagocytic Dysregulation	LCN2⁺ astrocytes engulf myelin debris (dMBP⁺)	Astrocytes in corpus callosum	Aberrant clearance of myelin fragments (non-ischemic areas)	LCN2 KO → ↑ myelin area, ↓ white matter injury score, ↑ myelin protein expression	(Wan et al., 2022)
BBB Disruption	Promotes endothelial ferroptosis; Modulates HMGB1/Nrf2/HO-1	Endothelial cells	Exacerbates BBB damage → secondary demyelination	Linked to ICH/TBI models	(Zhang et al., 2022, Liu et al., 2024)
Neuroinflammation & Cognitive Impairment	Interacts with inflammatory cytokines; ↓ NMDA receptor signaling	Neurons	Synaptic dysfunction & cognitive deficits	—	(Zhang et al., 2022, Kim et al., 2024)
Signaling Pathways	1. JAK2-STAT3 activation 2.HIF-1α-LCN2-VEGFα axis 3. PERK pathway activation	Multiple neural cells	Pro-apoptotic signaling; Angiogenesis dysregulation; ER stress amplification	Pharmacological pathway inhibitors show protection in preclinical models	(Chen et al., 2020, Fields, 2010)

dLCN2 (Lipocalin-2): A secreted protein sharply upregulated during the post-stroke inflammatory response, also known as neutrophil gelatinase-associated lipocalin. It functions as a key mediator of central nervous system injury. e24p3R/LRP2: The functional receptor for LCN2, mediating its endocytosis and subsequent activation of downstream signaling pathways.

#### 2.2.2 Mitochondria: the downstream effector

**Fig. 4:**
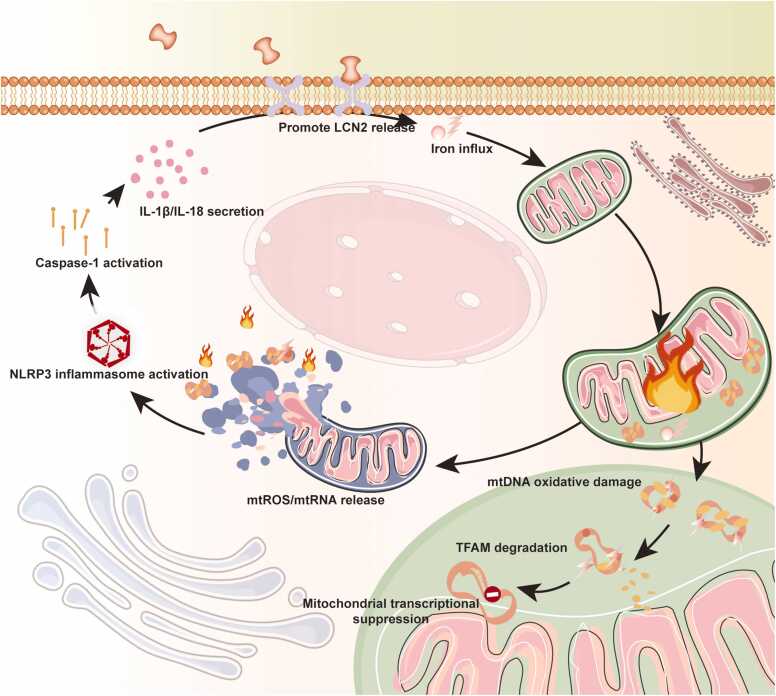
LCN2-mediated positive feedback loop of ferric ion-mitochondrial damage. Figure legend: LCN2 transports Fe²⁺ into astrocytes, leading to: (1) Mitochondrial ROS burst→ Oxidative damage of mtDNA→ TFAM degradation→ Impaired mitochondrial transcription; (2) Damaged mitochondria release mtROS and mtDNA→ Activate NLRP3 inflammasome via binding to NLRP3 sensor→ Caspase-1 cleavage→ Maturation of IL-1β/IL-18; (3) Secreted cytokines further promote LCN2 production, establishing a self-amplifying injury cycle. Abbreviations: TFAM: mitochondrial transcription factor A; 8-OHdG: 8-hydroxy-2′-deoxyguanosine; mtROS: mitochondrial reactive oxygen species.

LCN2 also exacerbates BBB damage by promoting endothelial ferroptosis and modulating the HMGB1/Nrf2/HO–1 pathway, which aggravates myelin loss (Liu et al., 2024). In addition, interactions between reactive microglia and oligodendrocytes via the CSF1/CSF1 receptor pathway contribute to oligodendrocyte ferroptosis and neurological deficits (Gu et al., 2024). In vitro, LCN2 treatment triggers the mitochondrial apoptotic pathway in oligodendrocytes: it reduces mitochondrial membrane potential, upregulates Bax, downregulates Bcl-2, and increases cytochrome c release (Chien et al., 2012). Electron microscopy reveals that during demyelination, excessive mitochondrial fission leads to swollen axons with disrupted cristae and fragmented oligodendrocyte mitochondria. This hyperfission state is marked by increased Drp1 phosphorylation at Ser616 and decreased Mfn2 levels (Wang et al., 2023b, Yang et al., 2025). High-resolution respirometry detected a 40 % decrease in mitochondrial Complex I activity and a 45 % reduction in ATP production in the corpus callosum 7 days after stroke. This was accompanied by a decreased NAD⁺/NADH ratio. Administration of nicotinamide riboside significantly increased brain NAD⁺ levels, restored ATP production nearly to baseline, and reduced the area of secondary callosal demyelination (Watson et al., 2014, Wang et al., 2022, Klaidman et al., 2003). After stroke, PINK1/Parkin-mediated mitophagy is initially activated to remove damaged mitochondria (Lei et al., 2021). However, LCN2-driven ROS production disrupts this by impairing autophagosome-lysosome fusion—evidenced by a higher LC3-II/LC3-I ratio and p62 accumulation—leading to blocked autophagic flux, which ultimately exacerbates mitochondrial damage and demyelination (Sultan et al., 2024). Mouse models show that impaired oxidative phosphorylation raises acetyl-CoA levels, initiating a cascade of astrocyte reactivity, neuronal oxidative stress, microglial activation, and impaired lipid synthesis for remyelination (Mi et al., 2023). At the core of this pathology, a “LCN2–iron–ROS–mitochondria” positive feedback loop has been identified as a key driver of secondary demyelination after stroke (Fig. 4).

#### 2.2.3 Additional pathways

Cholesterol metabolism dysregulation (Huang et al., 2025) and oxidative stress (Luo et al., 2024) are implicated in post-stroke demyelination. Interestingly, higher LDL-C appears to be protective, with a suggested dose–response relationship between cholesterol levels and demyelination severity (Wei et al., 2023). In hemorrhagic stroke, secondary demyelination is driven by microglial uptake of cholesterol/oxidized LDL via SR‑A1 and CD36, which activates the NLRP3 inflammasome (Zbesko et al., 2023, Sun et al., 2025).

Post-stroke secondary demyelination has recently been linked to histone deacetylase 3 (HDAC3) (Li et al., 2025b), which regulates the process by driving pro-inflammatory microglial proliferation (Zhang et al., 2024). Critically, HDAC3 acts as an epigenetic switch at the LCN2 promoter (Coutinho Costa et al., 2023). This connection informed a therapeutic strategy in MCAO mice: combined inhibition of HDAC3 (with RGFP966) and LCN2 (with a neutralizing antibody) successfully reduced demyelination. The approach, which combines acute phagocytosis inhibition with chronic LCN2/TLR4 blockade, may offer superior efficacy by addressing multiple injury pathways (Coutinho Costa et al., 2023).

## 3 Clinical and imaging markers

A consensus diagnostic criterion for post-stroke secondary demyelination is currently lacking. Two accessible biomarker categories are widely used in clinical practice: (1) Clinical–fluid biomarkers: Levels of serum neurofilament light chain (sNfL) and myelin basic protein (MBP) peak in correlation with demyelination volume in remote white matter regions (e.g., the corpus callosum). An sNfL level > 40 pg/ml can independently predict cognitive decline within 3 months post-stroke (Chitnis et al., 2024, Fung et al., 2025, Wang et al., 2021). (2) Imaging biomarkers: Advanced MRI techniques—diffusion tensor imaging (reduced fractional anisotropy), magnetization transfer ratio (decreased MTR), and myelin water imaging (reduced MWF)—can detect microstructural myelin damage. This detection occurs 2–4 weeks before T2 hyperintensity becomes visible on standard MRI (MacKay and Laule, 2016, McCreary et al., 2009). Furthermore, machine learning models that integrate FA and sNfL significantly improve the AUC for identifying secondary demyelination (Barro et al., 2022). A recent study identified the macromolecular proton fraction as a promising imaging biomarker for demyelination in ischemic stroke (Khodanovich et al., 2018). Together, these biomarkers provide a complementary “fluid–imaging” framework for dynamically monitoring demyelination burden and assessing early interventions. Importantly, post-stroke secondary demyelination is not merely a stroke complication but may also increase the risk for multiple sclerosis and is influenced by disordered cholesterol metabolism. Therefore, precise differential diagnosis and stratified management depend on integrating multimodal data from imaging, fluid biomarkers, and immune profiling.

## 4 Therapy and interventions

No standard therapy currently exists for post-stroke secondary demyelination. Recent research is primarily targeting three molecular pathways (Table 3).

**Table 3:** Emerging therapeutic strategies targeting post-stroke secondary demyelination.

Therapeutic axis	Intervention target/Agent	Molecular mechanism	Functional outcome	Experimental evidence	Reference
Astrocyte-Iron Axis Modulation	LCN2 inhibition	↓ LCN2-JAK2/STAT3 feedback loop; ↑ autophagy-lysosome pathway	↓ Neuroinflammation; ↓ oxidative stress; ↓ demyelination	LCN2 KO: ↑ myelin area, ↓ white matter injury	(Yuan et al., 2025, Yamazaki et al., 2025)
	Hydroxysafflor yellow A (HSYA)	Suppresses astrocytic LCN2 & JAK2/STAT3 signaling	↓ LCN2/inflammatory factors; ↑ neurological recovery	MCAO/R rats & OGD/R astrocytes: ↓ LCN2, ↓ IL-1β/TNF-α	(Li et al., 2022)
	PPARγ/Nrf2/γ-GCS activation	Enhances antioxidant pathway activity	↓ Microglial activation; ↓ ROS-mediated myelin damage	Preclinical models: reduced demyelination lesion volume	(Khodanovich et al., 2018)
Mitochondrial Repair	Hydrogen sulfide (H₂S)	Modulates RhoA pathway in oligodendrocytes	Promotes remyelination potential	In vitro OLs: improved mitochondrial function	(Jung and Ryu, 2023)
Remyelination Promotion	SIRT2 inhibition (AK-7)	Regulates Axl/PI3K/AKT-mediated microglial polarization	↑ M2 phenotype; ↓ white matter injury (WMI)	SAH models: improved neurofunction; ↑ OPC differentiation	(Lu and Wen, 2023)
	M2-EVsg	Directly targets Olig2⁺ oligodendrocytes	↑ White matter repair; ↑ myelin regeneration	Stroke models: enhanced OPC maturation & remyelination	(Yuan et al., 2025)
	Collagen I depletion	Blocks collagen-induced inhibition of oligodendrocyte differentiation	↑Remyelination efficiency; ↓microglial activation	LPC-induced demyelination mice: ↑ myelin thickness; ↓ axonal damage	(Li et al., 2022)
	Piezo1 channelh inhibition (GsMTx4)	Attenuates mechanosensitive cation channel activity	Reduces secondary neurodegeneration in late-phase demyelination	Demyelination disease models: ↓ axonal degeneration	(Yamazaki et al., 2025)

gM2 macrophage-derived Extracellular Vesicles: They directly deliver pro-regenerative signals to oligodendrocyte lineage cells, facilitating white matter repair.

hMechanosensitive ion channels activated by membrane stiffness or stretch.

First, targeting the astrocyte–iron axis may mitigate secondary demyelination progression. Inhibiting LCN2 or activating the PPARγ/Nrf2/γ‑GCS antioxidant pathway can reduce microglial activation and oxidative stress, thereby alleviating demyelinating injury (Chen et al., 2025). In a study using MCAO/reperfusion (R) rat and primary astrocyte OGD/R models, Song et al. showed that hydroxysafflor yellow A attenuates post-ischemic neuroinflammation and neurological deficits by inhibiting the LCN2–JAK2/STAT3 feedback loop in astrocytes. This inhibition reduces LCN2 and inflammatory cytokine expression, ameliorating inflammatory injury linked to secondary demyelination (Song et al., 2025). Additionally, recent studies suggest LCN2 is a target of the autophagy–lysosome pathway, indicating that autophagy activation could be a viable therapeutic strategy to lower LCN2 levels (Jung and Ryu, 2023).

Second, multiple molecular targets show potential. Hydrogen sulfide modulates oligodendrocyte RhoA to aid remyelination (Lu and Wen, 2023). SIRT2 regulates microglial polarization via Axl/PI3K/AKT, suggesting therapeutic value for AK-7 (Yuan et al., 2025). M2-derived extracellular vesicles may repair white matter by targeting Olig3 (Li et al., 2022). Type I collagen accumulation inhibits remyelination and is a novel target (Yamazaki et al., 2025). Finally, inhibiting the Piezo1 channel with GsMTx4 may alleviate late-stage neurodegeneration (Velasco-Estevez et al., 2020).

## 5 Conclusions and outlook

Post-stroke secondary demyelination is a progressive white matter injury that develops distal to the initial infarct. It is driven by a cascade of events involving dysregulated neuroglial interactions, mitochondrial dysfunction, and oxidative stress. Central to this pathology is a maladaptive interplay among astrocytes, microglia, and oligodendrocytes. Reactive astrocytes amplify damage through LCN2 secretion, hyperactive phagocytosis, and disrupted iron homeostasis. Concurrently, endoplasmic reticulum stress, mitochondrial failure, and excitotoxicity promote oligodendrocyte apoptosis, further compromising white matter integrity. Clinically, this process is linked to cognitive decline. Promising biomarkers for its detection and monitoring include sNfL and advanced MRI techniques such as diffusion tensor imaging, magnetization transfer ratio, and myelin water fraction.

Current therapeutic strategies focus on three key pathways: (1) targeting the LCN2–JAK2/STAT3 axis to mitigate astrocyte-mediated inflammation and iron dysregulation; (2) restoring mitochondrial function through NAD⁺ supplementation or enhanced mitophagy; and (3) promoting reparative microglial phenotypes and oligodendrocyte differentiation. Emerging approaches also aim to overcome remyelination barriers by inhibiting HDAC3, depleting type I collagen, or modulating Piezo1 channels.

Despite these advances, key challenges remain. The temporal dynamics and regional specificity of glial responses require further elucidation. Translational gaps persist in refining biomarkers for real-time tracking and validating therapeutic efficacy in aged models and human cohorts. Future studies should prioritize combinatorial strategies that concurrently address acute inflammatory triggers and chronic regenerative failure—for example, by dual targeting of LCN2 and HDAC3. Critical questions also warrant investigation, such as whether demyelination is a consequence or a cause of axonal injury, and whether peripheral neuropathy can trigger central demyelination via molecular mimicry or epitope spreading. Furthermore, exploring the roles of cholesterol metabolism and extracellular matrix remodeling may uncover novel therapeutic avenues. Ultimately, integrating multimodal biomarkers with targeted interventions holds promise for mitigating secondary demyelination and improving long-term functional outcomes after stroke.

## Funding

This research was supported by the following grants: the Joint Fund for Scientific Research in Public Hospitals Project (Grant No. 2024GLLH0481); the University-Level Education Reform Research and Practice Project of Inner Mongolia Medical University (Grant No. NYJXGGSJ2025024); and the Baotou City Health Science and Technology Project (Grant No. 2023wsjkkj05).

## CRediT authorship contribution statement

**Ruonan Cao:** Writing – original draft, Visualization. **Chaoran Liu:** Visualization. **Zhihui Liu:** Writing – review & editing, Funding acquisition. **Wenjing Ou:** Writing – review & editing, Methodology, Visualization.

## Declaration of Competing Interest

The authors declare that they have no known competing financial interests or personal relationships that could have appeared to influence the work reported in this paper.

## Data Availability

Data sharing not applicable to this article as no datasets were generated or analysed during the current study.
